# Bridging the gap: Introducing a socio-cultural dimension to explain beliefs about man-made threats

**DOI:** 10.1177/09636625221095723

**Published:** 2022-05-09

**Authors:** Isabella Glogger, Adam Shehata

**Affiliations:** University of Gothenburg, Sweden

**Keywords:** Antimicrobial resistance, belief gap hypothesis, climate change, media use, socio-cultural dimension of ideology

## Abstract

The belief gap hypothesis focuses on why individuals differ in beliefs about the causes and consequences of issues despite expert consensus. Offering ideological rationalization and media use as an explanation for diverting beliefs, it, so far, has focused on ideological priors that describe traditional socio-economic cleavages—even if scientific and environmental issues go beyond monetary questions. In this study, we aim to counter this shortcoming by introducing a socio-cultural dimension of ideology to research on the belief gap hypothesis. Comparing two issues of man-made threats—climate change and antimicrobial resistance—and emphasizing more strongly the role of media use for belief gaps, we find that a socio-cultural dimension of ideology serves as a better predictor for diverting beliefs about climate change but not for antimicrobial resistance. In contrast to left-leaning media, using right-leaning media outlets widens climate change belief gaps.

What people believe about the causes and consequences of environmental and scientific issues is crucial for the next generations—especially when medical, social, and economic futures are at stake and present-day human action is the root for these concerns. Climate change and antimicrobial resistance (AMR) are two examples of issues that are human-made and human-threatening at the same time. Recently, the United Nations declared AMR as a global health emergency that “will have disastrous impact within a generation” (United Nations, 2019: 4). The severity of climate change is described similarly (Atanasova and Koteyko, 2017). Although these issues come with a great extent of scientific consensus, public opinion seems to be less unified (for climate change, for example, for the United States: Leiserowitz et al., 2019; for Germany: Metag et al., 2017).

The belief gap hypothesis provides an explanation for why individuals can differ in their beliefs about societal issues (Hindman, 2009). Aiming to protect their identity, people strive to be in line with the beliefs held in the ideological group they associate with. As a result, belief gaps will emerge; that is, individuals of different ideologies will differ in their perceptions about societal issues. Such ideological belief gaps depend also on the media coverage of issues and the level of political issue contestation (Hindman, 2009). If an issue is politically contested and heavily reported by the media, the belief gap based on ideological rationalization becomes wider. Belief gaps have already been described for issues such as climate change (Hindman, 2009) or immigration (Saldaña et al., 2021).

Research on the belief gap hypothesis is, however, accompanied by several limitations. First, ideological rationalization has mainly been operationalized in terms of the left-right dimension, describing traditional socio-economic cleavages (Ford and Jennings, 2020). Environmental and scientific issues go beyond monetary aspects, however. They raise questions about health, inter-generation fairness, and resource equity from a global perspective and call for a broader socio-cultural outlook when addressing diverting public beliefs about them (O’Brien and Wolf, 2010). Second, the belief gap hypothesis rests on the premise that media use has the ability to widen belief gaps, but this assumption has rarely been tested empirically (for an exception, see Newman et al., 2018; Saldaña et al., 2021). The same holds for, third, the contestation of issues (Saldaña et al., 2021). Finally, most studies on the belief gap hypothesis were limited to the United States, whose political and media systems are not comparable with those, for example, in Sweden (Strömbäck and Dimitrova, 2006). Hence, little is known about the effect of media use on belief gaps in media systems that are less commercialized and colored by partisanship.

In this study, we aim to overcome these shortcomings by adding the socio-cultural dimension of ideology to explain belief gaps about man-made risk issues. This second dimension of political ideology reflects a recent shift toward post-materialistic values, such as multiculturalism, gender equality, and environmental protection in several western democracies in recent decades (Ford and Jennings, 2020). It, thus, (1) may be better suited to explain belief gaps on such issues than the traditional socio-economic left–right dimension. We, furthermore, test the two main assumptions of the belief gap hypothesis, namely, if (2) media use widens belief gaps and (3) gaps only emerge for contested, highly salient issues. To do so, we compare two issues of man-made threats—climate change and AMR—that are similar in scholarly evaluation but differ in media salience and contestation in political and public discourse. The study relies on a two-wave panel survey conducted in Sweden between February and November 2020, extending research on diverting beliefs beyond a US context.

## 1. Introducing a socio-cultural dimension of ideology to explain beliefs about man-made threat issues

Various theories—different in their focus and research tradition—aim to explain how people form beliefs. In this article, we focus on the belief gap hypothesis, which rests on the premise that people tend to exert directional motivated reasoning (Kunda, 1990) when forming and updating beliefs (Eveland and Cooper, 2013). People aim to be congruent with their prior values, ideologies, or worldviews—experienced through the perceived or actual affiliation with a group—to maintain this affiliation. In this sense, directional motivated reasoning is an identity-protective cognition (Druckman and McGrath, 2019).

Building on the long-standing tradition of the knowledge gap hypothesis (Tichenor et al., 1970), the belief gap hypothesis states that belief gaps about politically and publicly disputed issues emerge between people of different ideological leaning. The relationship between beliefs about an issue and ideology strengthens further—the gaps become wider—when media coverage about the issue increases. Hence, the belief gap hypothesis comprises the same basic principles as the knowledge gap hypothesis but modifies its renowned precursor by two aspects. First, it takes a non-positivistic stance on information. While knowledge, as operationalized in the knowledge gap research, is considered to be objective, beliefs are claims about reality that may or may not be true (Hindman, 2009). The second modification refers to ideological rationalization as one of the sources in the formation of beliefs. The knowledge gap hypothesis aims to explain the differences in knowledge by comparing socio-economic statuses of groups, for example, education. The belief gap hypothesis, in contrast, posits that (political) ideology is a better predictor. This is based on two rationales (Hindman, 2009): (1) in an environment of politicized and contested issues, those with political and economic power suggest which beliefs about these issues should be held; (2) individuals strive to be in line with their own values or the ones of the group they identify with, aiming to protect their social identity. Consequently, they update and hold beliefs depending on their prior preferences, such as ideology or partisanship.

Ideological rationalization in the form of left–right ideology has been found to serve as a better predictor for beliefs about scientific (risk) issues than education in the context of the belief gap hypothesis. Several studies on climate change confirmed this assumption (Häkkinen and Akrami, 2014; Hindman, 2009; Veenstra et al., 2014): individuals on the ideological left hold beliefs more in line with those of the scientific community than individuals on the ideological right—independently from education. When ideology was operationalized by partisanship, similar patterns were found (Lyons et al., 2018; Veenstra et al., 2014), also for other issues such as the origin of humankind (Veenstra et al., 2014) or fracking (Veenstra et al., 2016). In such studies, political ideology is typically described in terms of a left–right or liberal–conservative dimension (Jost et al., 2009). Traditionally, this dimension represents cleavages over socio-economic issues (Ford and Jennings, 2020). Scientific issues, such as climate change or AMR, are, however, not exclusively economic issues reflected in a monetary left–right cleavage. Rather, anthropogenic risk issues affect societies at large, triggering questions about values, for instance, about equality when it comes to shrinking resources of water, food, or land (O’Brien and Wolf, 2010).

While the belief gap hypothesis has so far fallen short of acknowledging the need to incorporate an ideological prior of this kind, the cultural cognition theory accounts for such calls by incorporating a broader cultural outlook to explain diverting beliefs (Kahan et al., 2011). While not regarding the role of news media use in the formation of diverting beliefs, the cultural cognition theory postulates that people form beliefs about scientific risk, evidence, and consensus in line with their worldviews. Worldviews comprises individuals’ preferences for how society is organized and for how social interactions with others should be characterized (Koltko-Rivera, 2004). In that sense, worldviews share similarities with political ideology but cover broader value dimensions (Newman et al., 2018). Several studies support the assumption that worldviews, by working as a special type of motivated reasoning (Kunda, 1990), help explain beliefs about environmental risk issues, such as polluted water runoff (Ahern et al., 2016), climate change (Diehl et al., 2019; Hornsey et al., 2016), or emerging technologies (Druckman and Bolsen, 2011; Kahan et al., 2009).

These findings underscore the importance of incorporating a broader cultural ideological outlook to the belief gap research, which goes beyond the traditional socio-economic left–right dimension of ideology. The socio-cultural dimension of ideology offers such an outlook. Understood as a second dimension of political ideology (Ford and Jennings, 2020), it reflects the shift from economic to post-materialistic values, such as multiculturalism, gender and generation equality, and environmental protection, as observed in several Western democracies in recent decades (Inglehart and Norris, 2017; Kriesi, 2010). As such, the socio-cultural dimension of ideology goes beyond the predominantly economic concerns of the socio-economic dimension of ideology and is, thus, suited for studying beliefs that pertain to environmental and public health questions.

## 2. The role of issue contestation: Comparing two man-made threat issues

Next to ideological rationalization, the belief gap hypothesis states that the degree of contestation plays an essential role in the formation of diverting beliefs (Hindman, 2009). Comparing the two man-made threats of climate change and AMR in terms of contestation is crucial to make claims about belief formation about these issues. We illustrate contestation by comparing scientific consensus, degree of politicization, public opinion, and media salience.

First, scientific consensus is similar for both issues, with little to no expert contestation on the causes and effects of climate change and AMR. Researchers agree on the severity of AMR in terms of being global threats to humanity—physically, socially, and economically (Laxminarayan et al., 2013). For climate change, the Intergovernmental Panel on Climate Change (IPCC), a UN body of climate experts, concluded that the increase in anthropogenic greenhouse gas emissions, such as carbon dioxide, “[is] extremely likely to have been the dominant cause of the observed warming since the mid-20th century” (IPCC, 2014: 4). Similarly, humans contributed tremendously to the resistance of bacteria and fungi to antibiotics, by the overuse and misuse of these medications (United Nations, 2019).

Second, differences between climate change and AMR can be found in terms of politicization, which “in general terms means the demand for or the act of transporting an issue into the field of politics—making previously unpolitical matters political” (Zürn, 2014: 50; for a recent discussion of the concept, see Palonen et al., 2019). In that sense, climate change is “the paradigmatic example of politicization” (Bolsen et al., 2014: 4). Chinn et al. (2020), for example, operationalized politicization by comparing the amount of references to scientific or political actors in US newspaper coverage on climate change, finding a decrease in references to scientists while the politicians became more prominent in climate change coverage. In contrast, Boklage and Lehmkuhl (2019), who content-analyzed the German news coverage about AMR over the course of 10 years, found that it was dominated by references to scientists—and that only a few political actors contribute to the AMR discourse.

Third, climate change and AMR differ in the degree to which they are established in public discourse. When in 1985 a hole in the ozone layer was discovered, terms such as greenhouse effect and later global warming and climate change became part of the public agenda (Bell, 1994). Reviewing surveys over the course of 20 years, Nisbet and Myers (2007) found that US citizens have become increasingly aware of climate change since the 1980s, with over 90% of survey participants having heard or read about the issue in the mid-2000s. Similar trends can be found in the European Union (European Commission, 2019). Far less established on the public agenda is AMR: Resistance to antibiotics was described some years after the discovery of antibiotics in the 1920s but only raised first concerns in the scientific community and among public health agencies in the 1980s (Podolsky, 2018). The general public remains largely uninformed about AMR: McCullough et al. (2016) concluded from a systematic review of studies on attitudes and beliefs about AMR that the “public have an incomplete understanding of and misperceptions about antibiotic resistance” (p. 31).

Fourth, media salience plays a crucial role in the formation of belief gaps. The literature describes differences in *how much* and *how* the media cover climate change and AMR. While climate change is prominently reported by the news media in many countries (Schmidt et al., 2013), AMR is a niche issue on the media agenda in, for example, Germany (Boklage and Lehmkuhl, 2019), Australia (Davis et al., 2020), or the United Kingdom and the United States (Singh et al., 2016). Analyzing climate change coverage in Germany, India, the United Kingdom, the United States, and Switzerland, Brüggemann and Engesser (2017) found climate skeptic voices in the media coverage overrepresented—despite scientific consensus. In contrast, uncertainty or a contrary position on the threat of AMR was rarely found in German news coverage (Boklage and Lehmkuhl, 2019).

## 3. The role of media use in belief gaps formation

The last factor that is crucial for the emergence of belief gaps is media use, but it has rarely been empirically addressed so far. Studies on the belief gap hypothesis either only point at the importance of incorporating news media use to the analysis (Hindman, 2009), include liberal and conservative media use as an additional predictor (Diercks and Landreville, 2017; Hindman and Yan, 2015; Saldaña et al., 2018, 2021; Veenstra et al., 2014), or assume changes in media coverage over time (Hindman, 2009, 2012; Hindman and Yan, 2015). These approaches, however, are insufficient for addressing whether belief gaps are related to communication (Nisbet, 2008). Understanding belief gaps as communication gaps means that ideological priors do not alone predict beliefs gap but that they *interact* with media use or attention. The assumption that media use interacts with ideological priors in the formation of belief gaps is backed up by the findings from knowledge gap hypothesis research (Gaziano, 2017; Lind and Boomgaarden, 2019). Also, the cultural cognition theory posits that individuals remember better and assign more importance to information that is salient in the group they share worldviews with while dismissing information not in line with their worldviews, resulting in belief reinforcement (Kahan, 2013).

A few studies have taken media use as a moderator into account. Nisbet et al. (2015) focused on attention to different types of media content, finding that attention to political news, scientific news, and entertainment TV interacted with liberal–conservative ideology. For example, conservatives were less likely to report correct beliefs about climate change when they were more attentive to political news. Carmichael et al. (2017) concluded from a time series analysis between 2001 and 2014 for the United States that media use strengthened people’s beliefs about climate change when they consumed media reporting about climate change congenial to their prior liberal or conservative ideology. Using an experimental approach, Kahan et al. (2009) analyzed how issue salience—operationalized by information about a risk issue given to participants—interacted with ideology in the formation of belief gaps. In the condition, in which participants did not receive further information about an unfamiliar risk issue, participants formed uniform beliefs—independently from their worldviews. The opposite was the case when participants were provided information about the issue, resulting in belief gaps between individuals of opposing worldviews.

## 4. The Swedish context

Empirically, this analysis focuses on the case of Sweden. Similar to several other European countries, the emergence of a socio-cultural dimension of politics has been evident in Swedish politics in the last decades. Issues such as environment and immigration have been on top of the agenda for several years—and the continued success of the right-wing nationalist party the Sweden Democrats among voters since entering parliament in 2010 marks a significant shift in Swedish politics (Oscarsson and Holmberg, 2020). The importance of these socio-cultural issues is also reflected in the news coverage of the last election in 2018, characterized by a larger focus on these issues than on those associated with economic questions (Håkansson, 2020). While the Swedish political system has historically been dominated by the established left–right divide, this dimension is increasingly challenged and complemented by a socio-cultural dimension—as reflected in the party system, among voters and the emergence of new media.

We can also distinguish the two man-made threats of interest in terms of perception and polarization in Swedish society. Although Swedish citizens are among the most knowledgeable about climate change and AMR in the European Union (European Commission, 2018), there are differences in how aware individuals are about these issues. When asked about the most important societal problem in Sweden in an open-answer format in 2018, 4% of the participants named climate change and 10% the broader category of environment, but no one mentioned AMR or synonymous issues (Rönnerstrand, 2018). In addition, climate change and AMR differ in contestation among the public. Although Sweden is by no means comparable to the public contestation of climate change in the United States (Chinn et al., 2020), Rönnerstrand (2018) found for the Swedish public in 2018 that the gap between the highly concerned about climate change on the left ideological pole and the highly concerned on the right pole was a difference of 43 percentage points. For AMR, in contrast, the difference was only 10 percentage points (Rönnerstrand, 2018).

**Figure 1.**
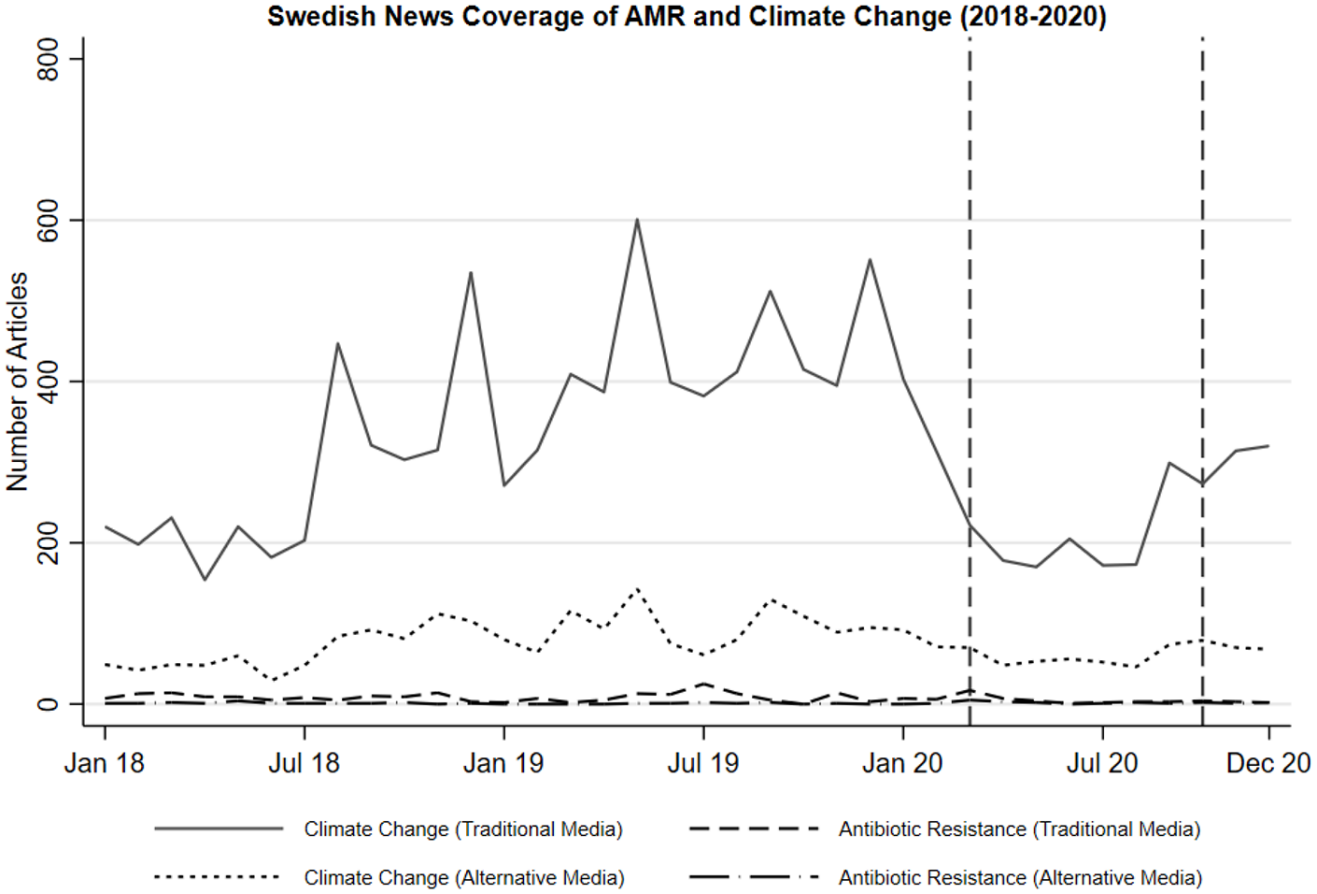
News coverage of AMR and climate change. *Note*. The number of news articles in five prominent traditional media outlets in Sweden (*Aftonbladet, Dagens Nyheter, Expressen, Svenska Dagbladet*, and *SVT Nyheter*) and six alternative online media outlets (*Aktuellt Fokus, Dagens Arena, ETC, Fria Tider, Nyheter idag*, and *Samhällsnytt*). Period of panel study marked with dashed lines (March–November 2020).

Climate change and AMR also differ vastly in Swedish media coverage. A longitudinal study comparing the two issues in several prominent news outlets showed that, while coverage of AMR was almost completely non-existent, climate change has received extensive reporting particularly since 2006 (Djerf-Pierre and Shehata, 2018). These differences are evident in Figure 1, which displays news reporting of the two issues between January 2018 and December 2020.^1^ Climate change is much more salient than antibiotic resistance—and this is particularly the case in traditional news media. Climate change reporting in Swedish alternative media is primarily driven by left-wing websites, but right-wing sources cover this topic as well. With respect to traditional news media, previous studies have documented almost universal acceptance of the dominant climate change frame in news reporting (Olausson, 2009; Shehata and Hopmann, 2012). Thus, we should expect that citizens who turn to traditional news media get a rather consensual view of climate change—at least on the news pages. Compared to the United States, the Swedish media system is significantly less polarized along ideological lines and political selective exposure plays relatively marginal role overall. With strong public service broadcasting institutions striving for neutrality and impartiality, Sweden lacks television and radio news channels with distinct political profiles. Instead, ideological news choices are limited to newspapers and political alternative media (Dahlgren et al., 2019) where editorial pages mirror distinct political affiliations. Furthermore, the emergence of political alternative media online has opened greater opportunities for ideological news choices.

Against this backdrop, Sweden provides an appropriate context to address our research questions. So far, the belief gap hypothesis has only been addressed in the United States where the political and media environment is substantially more polarized than in many European countries. At the same time, with new political cleavages emerging and opportunities for media choice growing, Sweden represents a “less-likely” context for identifying ideological belief gaps. Evidence supporting the belief gap hypothesis from the Swedish case would, therefore, also be likely to hold in more polarized political and media environments.

## 5. Research questions and hypotheses

Based on the presented theoretical and empirical findings, we pose several hypotheses and research questions. We, first, focus on the socio-cultural dimension of ideology as a prior to explain belief gaps. Based on calls for priors aligning with non-economic cleavages (O’Brien and Wolf, 2010), we suggest our first hypothesis:

*H1*. The relationship between the socio-cultural dimension of ideology and beliefs about climate change is stronger than between the socio-economic dimension of ideology and these beliefs.

Second, only when an issue is politically and publicly contested, we can observe gaps in beliefs between individuals of different ideological rationalization (Hindman, 2009). We, thus, ask the following:

*RQ1*. What is the relationship between ideological priors and beliefs about AMR as a non-contested issue?

Third, we aim to assess the role that media use plays in the formation of belief gaps. Supported by findings on the gap-widening effects of media use in the context of the knowledge gap hypothesis (e.g. Lind and Boomgaarden, 2019) and mechanisms of the cultural cognition theory (Kahan, 2012), we posit the following:

*H2*. The more ideologically consistent media people use, the wider are gaps in beliefs about climate change.

Finally, we contrast AMR as a low-salient issue with climate change as a high-salient issue in media coverage, asking the following question:

*RQ2*. How does ideologically consistent media use affect belief gaps about AMR based on ideological priors?

## 6. Method

To test the hypotheses and answer the research questions, we conducted a two-wave panel survey in Sweden. In this section, we describe our data, measures, and analytical approach.

### Data

We rely on a two-wave online panel study, collected between spring and fall 2020 (Wave 1: 17 March–21 April 2020; Wave 2: 26 October–20 November 2020). The data were collected online by the Laboratory of Opinion Research (LORE) at the University of Gothenburg, Sweden, using a probability sample of 3327 individuals, drawn from a panel with more than 75,000 people (for details on sample, see Table A1 in Supplemental Material). In Wave 1, 2171 started the survey and 2058 completed it (AAPOR RR5: 61.9%). The same sample was invited to participate in Wave 2. Out of the 3134 people who received the invite,^2^ 1785 started the survey and 1700 completed it (AAPOR RR5: 54.2%).

### Measures

#### Climate change and AMR beliefs

We used two statements to tap into what participants believed about the severity and the scientific evidence for climate change and AMR as dependent variables. Participants stated in Wave 2 to which degree they agreed to the following statements: (1) climate change/AMR is one of the greatest threats to humanity/to public health, and (2) scientific evidence for climate change/AMR is weak on a 7-point scale, reaching from 1 (“don’t agree at all”) to 7 (“totally agree”) (climate change threat: *M* = 5.61; *SD* = 1.69; climate change weak evidence: *M* = 2.14; *SD* = 1.53; AMR threat: *M* = 5.4; *SD* = 1.43; AMR weak evidence: *M* = 2.20; *SD* = 1.43).

#### Ideological priors

We used seven items to tap into participants’ ideology by assessing their attitudes toward various economic and socio-cultural policy proposals in Sweden. We asked the participants to rate the following proposals, which were inspired by Hooghe et al. (2002), ranging from 1 (“very good proposal”) to 5 (“very bad proposal”): Sweden should (1) reduce taxes (reversed); (2) accept fewer refugees (reversed); (3) introduce much harsher prison sentences for criminals (reversed); (4) aim for a multicultural society; (5) raise unemployment benefits; (6) reduce income disparities in society; and (7) not allow distribution of profits within state financed healthcare, schools, or other public services. We conducted an exploratory factor analysis, obtained by principal axis factoring and promax rotation, resulting in a two-factor solution. Since the first item loaded equally on both factors, we excluded it before conducting another factor analysis. Again, a two-factor solution was found (Kaiser–Meyer–Olkin (KMO) test of the sampling adequacy = .72; Bartlett’s test of sphericity: χ^2^ = 2341.625, *df* = 15, *p* < .001) (Table A2 in Supplemental Material). We labeled the socio-cultural dimension *GAL-TAN ideology*, ranging from “Green-Alternative-Liberal” (GAL) to “Traditional-Authoritarian-Nationalist” (TAN), and the traditional economic dimension *left–right ideology* (Hooghe et al., 2002).^3^ We combined the items of the factors into indices and rescaled them to range from 0 (GAL/left) to 1 (TAN/right) (GAL-TAN: *M* = 0.59, *SD* = 0.26, Cronbach’s α = .77; left–right: *M* = 0.34; *SD* = 0.22, Cronbach’s α = .63).

#### Media use

We constructed a media use variable that reflected both exposure to news and the political leaning of the outlets. Media use was measured in Wave 2 by asking the participants how often they had used 10 mainstream newspapers (online and offline) and 6 left- and 6 right-alternative online outlets in the 4 weeks prior to the measurements (from 1 (“never”), 2 (“seldom”), 3 (“1–2 days per week”), 4 (“3–4 days per week”), 5 (“5–6 days per week”), to 6 (“daily”)). We then weighted the participants’ individual media use by the outlets’ political leaning position (Table A3 in Supplemental Material). To reflect the difference between traditional newspapers’ clear distinction between news and views, where political affiliations are primarily found on the editorial pages and not in the news section, on the one hand, and the more salient political leaning of political alternative media, on the other hand, use of these outlets was weighted differently. Left-alternative media were weighted with −2, left-traditional media with −1, right-traditional with +1, and right-alternative with +2. Finally, we built an index, which we rescaled to range from 0 (“a predominantly left-leaning media diet”) to 1 (“predominantly right-leaning media diet”) (*M* = 0.28, *SD* = 0.09; Cronbach’s α = .82).

#### Control variables

We controlled for prior beliefs about climate change and AMR in Wave 1, using the same items as described above. We rescaled them to range from 0 to 1 (climate change threat: *M* = 0.73; *SD* = 0.30; climate change weak evidence: *M* = 0.21; *SD* = 0.27; AMR threat: *M* = 0.74; *SD* = 0.24; AMR weak evidence: *M* = 0.74; *SD* = 0.24). In addition, we controlled for gender, age, and education due to their described relationship with beliefs about scientific issues (Hornsey et al., 2016). The variable for gender was coded 0 (female, 50%) and 1 (male, 50%). Age was measured in six categories (under 30, 30–39, 40–49, 50–59, 60–69, and 70 years or above; rescaled to range from 0 to 1 (*M* = 0.54, *SD* = 0.33)); education in four categories (0 = “up to 9 years of schooling” (5%), 1 = “up to 12 years of schooling” (37%), 2 = “12 years and vocational training” (17%), 3 = “12 years and university degree” (41%)). We measured the control variables in Wave 1.

### Data analysis

To test our hypotheses and answer the research questions, we conducted a series of multiple linear regressions. To assess the influence of media use on the relationship between ideological priors and beliefs about climate change and AMR, we additionally included respective interaction terms to the regression models (H2 and RQ2). We make use of the advantages of our panel data by having all independent and control variables—including prior beliefs about AMR and climate change—measured in Wave 1, while the dependent belief variables were measured in Wave 2. By controlling for the lagged dependent variable, these autoregressive models capture the effects of our independent variables on *changes* in AMR and climate change beliefs over time (Finkel, 2008).

## 7. Results

To test our first hypothesis that poses that the socio-cultural dimension of ideology is a stronger predictor than the left–right dimension for beliefs about climate change, we conducted a series of multiple linear regressions (Table 1), controlling for age, gender, education, and prior beliefs about climate change and using cluster-robust standard errors.

**Table 1.** Multiple linear regressions of two dimensions of ideology on climate change and AMR beliefs.

	Model 1^a^	Model 2^b^	Model 3^c^	Model 4^d^
Constant	3.81***	1.20***	3.50***	1.82***
	(0.24)	(0.22)	(0.23)	(0.24)
Gender^e^	−0.17**	0.02	0.10	−0.06
	(0.06)	(0.07)	(0.07)	(0.07)
Age	−0.06	0.17	0.06	0.17
	(0.10)	(0.10)	(0.11)	(0.11)
Education^f^				
Medium low	0.20	−0.36	0.16	−0.26
	(0.16)	(0.21)	(0.16)	(0.21)
Medium	0.07	−0.37	0.24	−0.38
	(0.17)	(0.22)	(0.17)	(0.22)
High	0.09	−0.47*	0.26	−0.51*
	(0.16)	(0.21)	(0.16)	(0.21)
CC belief threat (*t*−1)	3.37***			
	(0.16)			
CC belief evidence (*t*−1)		2.63***		
		(0.19)		
AMR belief threat (*t*−1)			2.32***	
			(0.17)	
AMR belief evidence (*t*−1)				2.13***
				(0.18)
Socio-cultural dimension (GAL-TAN)	−0.92***	1.11***	0.09	0.56***
	(0.15)	(0.15)	(0.15)	(0.14)
Socio-economic dimension (left–right)	−0.33	0.09	−0.44*	−0.21
	(0.17)	(0.16)	(0.18)	(0.16)
*N*	1488	1483	1465	1449
*R* ^2^	.48	.37	.16	.19

*Note*. Unstandardized regression coefficients displayed. Robust standard errors in parentheses. All dependent variables were measured on a scale from 1 (*don’t agree at all*) to 7 (*totally agree*). All independent and control variables were rescaled to range from 0 to 1. GAL-TAN = socio-cultural dimension of ideology (“Green-Alternative-Liberal” (GAL) to “Traditional-Authoritarian-Nationalist” (TAN)). CC: Climate change; AMR: Antimicrobial resistance.

a Dependent variable: Agreement to the statement: Climate change is one of the greatest threats to humanity.

b Dependent variable: Agreement to the statement: Scientific evidence for climate change is weak.

c Dependent variable: Agreement to the statement: AMR is one of the greatest threats to public health.

d Dependent variable: Agreement to the statement: Scientific evidence for AMR is weak.

e Reference group: female.

f Reference group: low education.

*** *p* < .001; ** *p* < .01; * *p* < .05.

We found that the socio-cultural dimension of ideology (GAL-TAN) predicted the belief that climate change is one of the biggest threats to humanity. The more socio-culturally conservative (TAN) participants were (*B* = −0.92; *p* < .001), the less they believed in the threat for humans due to climate change (Model 1). The socio-cultural dimension of ideology also predicted the belief in the weakness of scientific evidence for climate change. The more socio-culturally conservative (TAN) participants were, the more they agreed to this statement (*B* = 1.11; *p* < .001) (Model 2); the socio-economic dimension (left–right) had no significant effect on either climate change belief, confirming the first hypothesis.

Our first research question RQ1 aimed to assess the influence of both dimensions of ideology on beliefs about AMR. Again, we conducted two multiple linear regression analyses, controlling for age, gender, education, and prior beliefs about AMR (Table 1, Models 3 and 4). Only socio-economic dimension (left–right) predicted significantly the belief that AMR poses one of the biggest threats to public health. The more right participants were, the less they believed in the threat of AMR (*B* = −0.44; *p* = .01). The belief about the weakness of scientific evidence for AMR, in contrast, was only predicted by the socio-cultural dimension of ideology (GAL-TAN), with participants scoring higher on the scale believing more in the weakness of AMR evidence (*B* = 0.56; *p* < .001). In comparison with beliefs about climate change, the answer to the first research question is less straightforward; both dimensions of ideology seem to influence beliefs about AMR, depending on the type of belief assessed.

**Figure 2.**
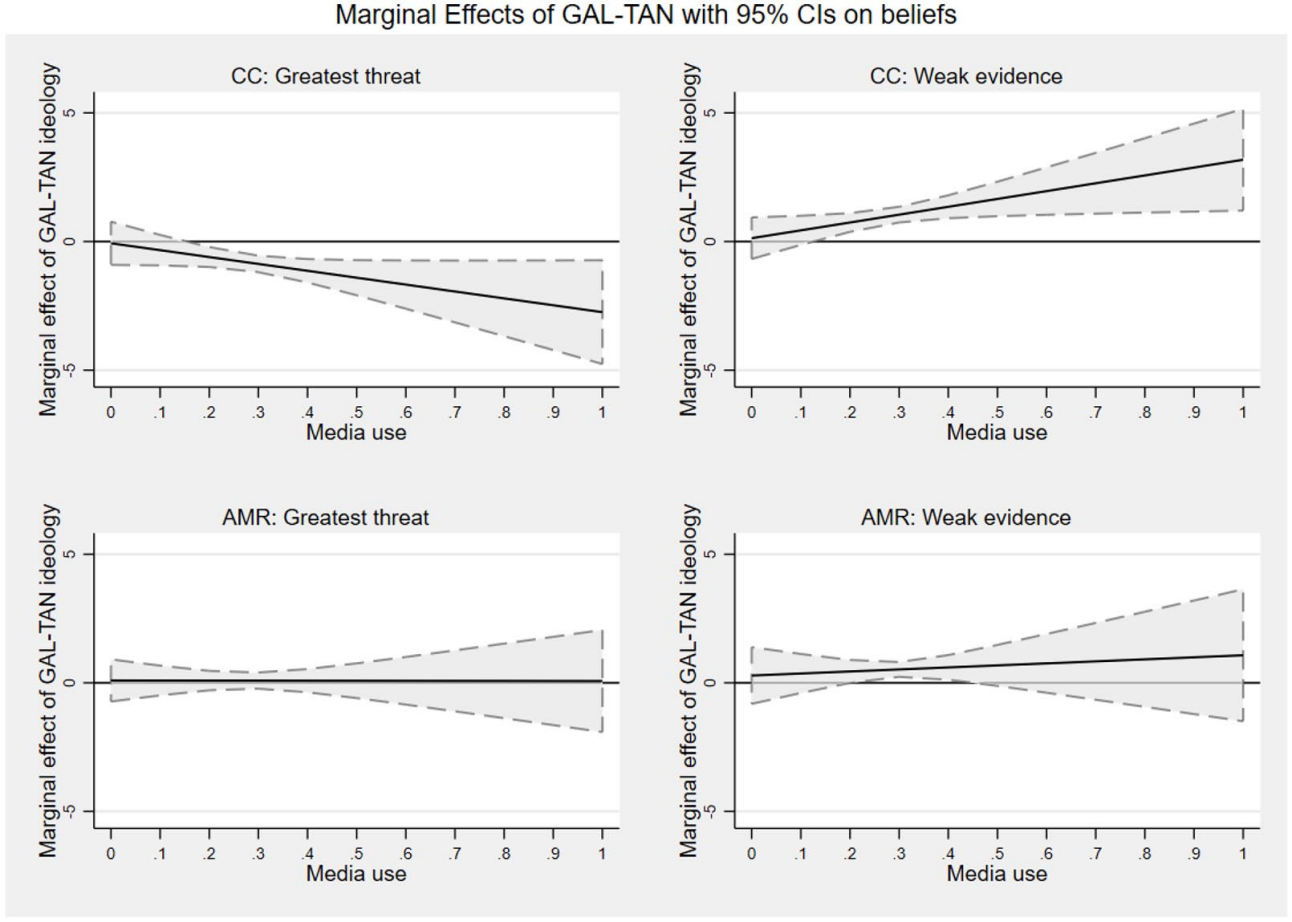
Marginal effects of socio-cultural dimension of ideology (GAL-TAN) on climate change and AMR beliefs in dependence of media use. *Note*. CC: climate change; AMR: antimicrobial resistance. Media use from left-leaning media use (0) to right-leaning media use (1). GAL-TAN = socio-cultural dimension of ideology (“Green-Alternative-Liberal” (GAL) to “Traditional-Authoritarian-Nationalist” (TAN)).

Our second hypothesis stated that the relationship between the socio-cultural dimension of ideology (GAL-TAN) and beliefs about climate change varies as a function of media use. We conducted two regressions with cluster-robust standard errors to assess the moderating effect of media use on the relationship between the socio-cultural dimension and two beliefs about climate change, controlling for age, gender, and prior beliefs (Figure 2; for detailed results, see Table A4 in Supplemental Material, Models 1b and 2b).

Figure 2 illustrates both interactions, showing how the marginal effect of the socio-cultural dimension (GAL-TAN) on beliefs changes with media use changing from left-leaning to right-leaning outlets. Following the approach by Brambor et al. (2006) for inspecting interactions, we see that the effect of GAL-TAN ideology becomes significant when participants use more right-leaning media. This is the case for both climate change beliefs. We thus confirm Hypothesis 2.

The second research question (RQ2) asked whether media use also influenced beliefs about AMR as a non-contested, non-salient issue in media coverage. Again, using multiple linear regression with cluster-robust standard errors and controlling for age, education, and prior beliefs about AMR, we did not find significant interaction effects between media use and GAL-TAN ideology on both AMR beliefs, as can be seen in Figure 1 (Table A5 in Supplemental Material, Models 1b and 2b).^4^

## 8. Discussion

The advent of societal issues that go beyond traditional socio-economic cleavages calls for integrating the socio-cultural dimension of ideology to research on the belief gap hypothesis. While previous studies built on left–right ideology or partisanship to explain belief gaps (e.g. Hindman, 2009, 2012; Newman et al., 2018), the purpose of our study was to introduce a second, socio-cultural dimension of ideology to belief gap research. This, we argued, seems more appropriate to address beliefs about scientific and environmental issues. Relying on a two-wave panel study conducted in Sweden and analyzing climate change and AMR, we furthermore aimed to study belief gaps in a non-US context and to compare issues of different degrees of contestation and media salience.

Our findings support several of our hypotheses and provide answers to our research questions. First, the socio-cultural dimension of ideology, indeed, is a better predictor than socio-economic left–right ideology for beliefs about climate change and for one belief about AMR. We decided to operationalize the socio-cultural dimension of ideology as GAL-TAN ideology (Hooghe et al., 2002), based on the issues we focused on and the country, in which the study was conducted. Similar considerations could also guide future research on belief gaps, with paying attention to the appropriateness of priors depending on issues and contexts. Several authors have offered conceptualizations of the socio-cultural dimension of ideology (for an overview, see Ford and Jennings, 2020). Second, our results showed that media use had the ability to widen belief gaps about climate change: When individuals identify more with the “TAN” end of the socio-cultural dimension and use more right-leaning media, they believe less in the danger of climate change and are more doubtful in terms of scientific evidence. In contrast, media use had no effect on the relationship between ideology and beliefs about AMR. Both findings are in line with the assumptions of the belief gap hypothesis (Hindman, 2009), underlining the role of ideological priors *and* media use in the formation of belief gaps. Third, our study also enabled us to compare two issues of man-made threats with varying degree of contestation. As stated by the belief gap hypothesis, belief gaps are most likely to occur when issues are contested. The fact that neither of the two ideological dimensions dominated as a predictor of AMR beliefs speaks to the core assumption of the belief gap hypothesis. Since research on belief gaps, which compares issues that differ in terms of contestation and salience, is still in its infancy (Saldaña et al., 2021), future studies should extend this approach. Since AMR is not a completely new issue in Sweden, future studies could focus on comparing newly emerging and well-established issues.

These results are not only of empirical merit for the studied issues and specific beliefs, but they also advance the belief gap hypothesis theoretically. On the one hand, the findings emphasize that the selection of ideological priors matters, specifying the belief gap hypothesis with regard to the mechanisms at work. As once Hindman (2009, 2012) argued that political ideology is more appropriate than socio-economic status when addressing beliefs instead of knowledge as analyzed in knowledge gap research, we argued that the selection of priors should be guided by the nature of the studied issues and the study context to increase the predictive power of the belief gap hypothesis. On the other hand, comparing a salient and contested to a low-salient and less contested issue brought the belief gap hypothesis forward theoretically. While it at first seems counterintuitive to study belief gaps on an issue that does not fulfill any of the priors to explain gaps, it enabled us to test the assumptions of the belief gap hypothesis in a most-likely and least-likely case. If we had found a moderating effect of media use on ideological rationalization and beliefs about a non-salient issue—such as AMR—the belief-gap-widening effect of media use as proposed by the belief gap hypothesis would have been challenged. In the same vein, finding different ideological priors to be at work for beliefs about a non-contested issue in contrast to beliefs about a highly politicized and contested issue strengthens the assumptions of the belief gap hypothesis: when issues leave the scientific realm and become of object of contestation and uncertainty (Bolsen et al., 2014), perceptions become more clearly colored by socio-cultural ideology.

At this point, limitations should not remain unmentioned. Although we were interested in the influence of media use on the relationship between ideology and beliefs about man-made threats, we relied on a broad classification of media outlets along the left–right ideological dimension. First, while we were able to assess the mere amount of media coverage, this approach does not provide information on whether the reporting on climate change and AMR differed in tone. Second, research on media coverage of socio-cultural issues is only in its infancy. Therefore, we were not able to distinguish the outlets along a socio-cultural dimension; instead of placing the outlets’ ideological leaning on a socio-cultural (GAL-TAN) scale, we thus decided to use the more established traditional left–right dimension. In future research, content analyses of the media coverage would help to overcome both limitations. The third limitation addresses the selection of issues in our study. By focusing on man-made threat issues, the generalizability of our findings is limited. Future studies may follow our approach and compare beliefs about issues of different contestation and media salience from other societal areas.

Even though our study comes with these limitations, it has opened research on the belief gap hypothesis to ideological priors that are more suited for current societal issues, referring to more than economic divisions. Focusing on such socio-cultural priors and addressing more profoundly the role of media use, will allow researchers to gain a better understanding of beliefs about the causes and consequences of issues that come with high costs for future generations.

## Supplemental Material

sj-docx-1-pus-10.1177_09636625221095723 – Supplemental material for Bridging the gap: Introducing a socio-cultural dimension to explain beliefs about man-made threats Click here for additional data file. Supplemental material, sj-docx-1-pus-10.1177_09636625221095723 for Bridging the gap: Introducing a socio-cultural dimension to explain beliefs about man-made threats by Isabella Glogger and Adam Shehata in Public Understanding of Science

## Notes

1. We conducted a keyword search in a news archive Mediearkivet, including five prominent traditional media outlets in Sweden (*Aftonbladet, Dagens Nyheter, Expressen, Svenska Dagbladet*, and *SVT Nyheter*) and six alternative online media outlets (*Aktuellt Fokus, Dagens Arena, ETC, Fria Tider, Nyheter idag*, and *Samhällsnytt*) to assess the number of climate change and antimicrobial resistance (AMR) articles (for exact procedure, see Supplemental Material).

2. In Wave 2, the sample was smaller due to complete dropouts from the standing panel or technical problems to reach participants.

3. Citizens and parties, who identify with the GAL-end of the scale support, among others, a great degree of personal freedom, gay and minority rights, multiculturalism, and environmental protection. Those at the TAN-end emphasize the importance of traditional family models, church, and the sovereignty of national states, for example (Hooghe et al., 2002). The importance of GAL-TAN issues has increased in Swedish recent politics, for example, for voters’ party choice (Oscarsson and Holmberg, 2020).

4. To assess the sensitivity of our findings to different classifications of the separate outlets, we replicated the interaction models using alternative weighting procedures for the media index. On the one hand, we constructed a media diet index using only −1 (left) and +1 (right) weights for different outlets. On the other hand, we also used a weighting procedure based on the ideological audience profile of each news outlet (see Fletcher et al., 2020). Outlets with a left-wing audience profile received a negative weighting score, while outlets with right-wing profile received a positive score, in proportion to the strength of the relationship between ideology and use of the specific outlet. The overall findings presented in Figure 2 were very similar across these alternative weighting procedures.
