# Supplementary material for: Bridging the gap: Introducing a socio-cultural dimension to explain beliefs about man-made threats
Source: Public Underst Sci. 2022 May 9;31(8):1063–78. doi: 10.1177/09636625221095723 (PMC9630961; doi:10.1177/09636625221095723)
Supplement: sj-docx-1-pus-10.1177_09636625221095723 – Supplemental material for Bridging the gap: Introducing a socio-cultural dimension to explain beliefs about man-made threats [file sj-docx-1-pus-10.1177_09636625221095723.docx]

**Supplemental material: Bridging the Gap: Introducing a Socio-Cultural Dimension to Explain Beliefs about Man-Made Threats**

**Isabella Glogger**

Department of Journalism, Media and Communication

UNIVERSITY OF GOTHENBURG

Sweden

Orcid: 0000-0002-3371-0485

**Adam Shehata**

Department of Journalism, Media and Communication

UNIVERSITY OF GOTHENBURG

Sweden

Table of Contents

Search strings for key word search in news archive2

Table A 1: Sample characteristics3

Table A 2: Factor loadings after explanatory factor analysis 5

Table A 3: Ideological leaning of outlets 6

Table A 4: Interaction effect of media use and socio-cultural dimension (GAL-TAN) of ideology on climate change beliefs 7

Table A 5: Interaction effect of media use and socio-cultural dimension (GAL-TAN) of ideology on AMR beliefs8

# Search strings for key word search in news archive

1. Climate Change (translation in parentheses)

klimatförändring* (*climate change*) OR klimatförhandling* (*climate negotiation*) OR koldioxidutsläpp* (*carbon dioxide emission*) OR klimathot* (*climate threat*) OR global uppvärmning* (*global warming*) OR växthuseffekt* (*greenhouse effect*) OR klimatavtal* (*climate agreement*) OR klimatfråg* (*climate issue*) OR klimatpolitik* (*climate politics*) OR Kyotoavtal* (*Kyoto protocol*) OR växthusgas*(*greenhouse gas*) OR klimatmöte* (*climate summit*) OR klimatpåverkan* (*climate impact*) OR klimatmål* (*climate targets*) OR utsläppsrätt* (*climate lawes*)

1. AMR (translation in parentheses)

“MRSA” OR “antibiotikaresistens*” (*antibiotic resistance*) OR (resistent* (*resistance*) NEAR/3 bakterier* (*bacteria*)) OR (resistens* (*resistance*) NEAR/3 antibiotika (*antibiotics*)) OR “multiresistenta bakterier” (*multi-resistant bacteria*) OR “ESBL” OR “superbakteri*” (*superbug*)

# Table A 1

*Sample characteristics*

|  |  | *N* |  | *Percent* |
| --- | --- | --- | --- | --- |
| *Gender* | |  |  |  |
|  | Female | 1,007 |  | 49.51 |
|  | Male | 1,027 |  | 50.49 |
|  |  |  |  |  |
| *Age* | |  |  |  |
|  | Under 30 | 232 |  | 11.41 |
|  | 30-39 | 291 |  | 14.31 |
|  | 40-49 | 378 |  | 18.58 |
|  | 50-59 | 363 |  | 17.85 |
|  | 60-69 | 410 |  | 20.16 |
|  | 70 or above | 360 |  | 17.7 |
|  |  |  |  |  |
| *Education* | |  |  |  |
|  | up to nine years of schooling | 105 |  | 5.16 |
|  | up to nine years of schooling | 685 |  | 33.68 |
|  | 12 years and vocational training | 323 |  | 15.88 |
|  | 12 years and university degree | 765 |  | 37.61 |
|  |  |  |  |  |
| *Income ^a^* | |  |  |  |
|  | less than 4 000 | 80 |  | 3.5 |
|  | 4,000-8,999 | 80 |  | 3.5 |
|  | 9,000-12,999 | 150 |  | 6.56 |
|  | 13,000-15,999 | 145 |  | 6.34 |
|  | 16,000-18,999 | 114 |  | 4.98 |
|  | 19,000-22,999 | 166 |  | 7.26 |
|  | 23,000-25,999 | 155 |  | 6.78 |
|  | 26,000-29,999 | 250 |  | 10.93 |
|  | 30,000-36,999 | 393 |  | 17.18 |
|  | 37,000-44,999 | 278 |  | 12.16 |
|  | 45,000-54,999 | 163 |  | 7.13 |
|  | 55,000-64,999 | 53 |  | 2.32 |
|  | more than 65,000 | 55 |  | 2.4 |
|  | Don't know/don't want to answer | 59 |  | 2.58 |
|  | Others | 11 |  | 0.48 |
|  |  |  |  |  |
|  |  |  |  |  |
|  |  |  |  |  |
|  |  |  |  |  |
|  |  |  |  |  |
| Table A1 continued | |  |  |  |
|  | |  |  |  |
|  | | *N* |  | Percent |
| *Employment* | |  |  |  |
|  | Full-time employed | 1027 |  | 44.91 |
|  | Part-time employment | 142 |  | 6.21 |
|  | Self-employed | 133 |  | 5.82 |
|  | Unemployment training scheme | 10 |  | 0.44 |
|  | Unemployed | 44 |  | 1.92 |
|  | Retired | 523 |  | 22.87 |
|  | On sick leave | 56 |  | 2.45 |
|  | Student | 161 |  | 7.04 |
|  | Others | 71 |  | 3.1 |
|  |  |  |  |  |
|  |  | *N* |  | *M (SD)* |
| Political interest ^b^ | | 2,031 |  | 2.05 (0.7) |
|  |  |  |  |  |

*Note*. a) Income in Swedish Krona (SEK); b) measured on a scale from 1 = “very interested” to 4 “not interested at all”.

# Table A 2

# *Factor loadings after explanatory factor analysis, obtained by principal axis factoring and promax rotation*

|  | Factor 1 | Factor 2 | Uniqueness |
| --- | --- | --- | --- |
| Sweden should… |  |  |  |
| accept fewer refugees | **0.89** | 0.01 | 0.20 |
| introduce tougher prison sentences for criminals | **0.82** | -0.11 | 0.38 |
| aim for a multicultural society | **0.74** | 0.16 | 0.34 |
|  |  |  |  |
| not allow distribution of profits within stately financed healthcare, schools or other public services | -0.12 | **0.82** | 0.38 |
| reduce income disparities in society | 0.16 | **0.75** | 0.33 |
| raise unemployment benefits | 0.01 | **0.72** | 0.48 |
| Eigenvalue | 2.6692 | 1.2268 |  |
| Variance explained (in%) | 45 | 21 |  |

# Table A 3

# *Ideological leaning of outlets*

| Outlet | Type and leaning |
| --- | --- |
| Aftonbadet (print) | Mainstream left-leaning |
| Aftonbladet (online) | Mainstream left-leaning |
| Aktuellt Fokus | Alternative left-leaning |
| Aktuellt Politiken | Alternative left-leaning |
| Arbetet | Alternative left-leaning |
| Dagens indusri (online) | Mainstream right-leaning |
| Dagens industi (print) | Mainstream right-leaning |
| Dagens Nyheter (print) | Mainstream right-leaning |
| Dagens Nyheter (online) | Mainstream right-leaning |
| DagensArena | Alternative left-leaning |
| ETC | Alternative left-leaning |
| Expressen (online) | Mainstream right-leaning |
| Expressen (print) | Mainstream right-leaning |
| Feministisktperspektiv | Alternative left-leaning |
| FriaTider | Alternative right-leaning |
| Ledarsidorna | Alternative right-leaning |
| Nya Tider | Alternative right-leaning |
| Nyheter Idag | Alternative right-leaning |
| Samhällsnytt | Alternative right-leaning |
| Samtiden | Alternative right-leaning |
| Svenska Dagbladet (online) | Mainstream right-leaning |
| Svenska Dagbladet (print) | Mainstream right-leaning |

*Note*. Leaning of outlets based on their own ideological identification.

# Table A 4

# *Interaction effect of media use and socio-cultural dimension (GAL-TAN) of ideology on climate change beliefs*

|  | Model 1a ^a^ | Model 1b ^a^ |  | Model 2a^b^ | Model 2b^b^ |
| --- | --- | --- | --- | --- | --- |
| Constant | 3.74*** | 3.24*** |  | 1.09*** | 1.67*** |
|  | (0.31) | (0.39) |  | (0.29) | (0.39) |
|  |  |  |  |  |  |
| Gender ^c^ | -0.16* | -0.15* |  | 0.07 | 0.06 |
|  | (0.07) | (0.07) |  | (0.07) | (0.07) |
| Age | -0.12 | -0.12 |  | 0.22* | 0.23* |
|  | (0.11) | (0.11) |  | (0.11) | (0.11) |
| *Education* ^d^ |  |  |  |  |  |
| Medium low | 0.21 | 0.21 |  | -0.37 | -0.36 |
|  | (0.18) | (0.18) |  | (0.24) | (0.24) |
| Medium | 0.20 | 0.20 |  | -0.38 | -0.38 |
|  | (0.20) | (0.19) |  | (0.25) | (0.25) |
| High | 0.17 | 0.16 |  | -0.48 | -0.47 |
|  | (0.18) | (0.18) |  | (0.25) | (0.24) |
| CC belief: threat (t-1) | 3.47*** | 3.45*** |  |  |  |
|  | (0.17) | (0.18) |  |  |  |
| CC belief: evidence (t-1) |  |  |  | 2.74*** | 2.72*** |
|  |  |  |  | (0.21) | (0.21) |
|  |  |  |  |  |  |
| Socio-cultural dimension (GAL-TAN) | -0.81*** | -0.07 |  | 0.98*** | 0.13 |
|  | (0.16) | (0.43) |  | (0.15) | (0.41) |
| Socio-economic dimension (left-right) | -0.41* | -0.45* |  | 0.12 | 0.16 |
|  | (0.19) | (0.19) |  | (0.18) | (0.18) |
|  |  |  |  |  |  |
| Media use | -0.37 | 1.60 |  | 0.41 | -1.81 |
|  | (0.45) | (1.03) |  | (0.49) | (1.06) |
| GAL-TAN x media use | | -2.68 |  |  | 3.05* |
|  |  | (1.41) |  |  | (1.38) |
|  |  |  |  |  |  |
| *N* | 1,265 | 1,265 |  | 1,264 | 1,264 |
| *R^2^* | 0.50 | 0.50 |  | 0.38 | 0.38 |

*Note*. Unstandardized regression coefficients displayed. Robust standard errors in parentheses. All independent and control variables were rescaled to rage from 0 to 1. GAL-TAN = socio-cultural dimension of ideology (“Green-Alternative-Liberal” (GAL) to “Traditional-Authoritarian-Nationalist” (TAN)). CC = climate change.

a Dependent variable: Agreement to the statement: Climate change is one of the greatest threats to humanity.

b Dependent variable: Agreement to the statement: Scientific evidence for climate change is weak.

c Reference group: female.

d Reference group: low education.

*** *p*<0.001, ** *p*<0.01, * *p*<0.05

# Table A 5

# *Interaction effect of media use and socio-cultural dimension (GAL-TAN) of ideology on AMR beliefs*

|  | Model 1a ^a^ | Model 1b ^a^ |  | Model 2a ^b^ | Model 2b *b* |
| --- | --- | --- | --- | --- | --- |
| Constant | 3.41*** | 3.40*** |  | 1.64*** | 1.79*** |
|  | (0.26) | (0.37) |  | (0.28) | (0.47) |
|  |  |  |  |  |  |
| Gender ^c^ | 0.06 | 0.06 |  | -0.04 | -0.04 |
|  | (0.07) | (0.07) |  | (0.07) | (0.07) |
| Age | 0.09 | 0.09 |  | 0.15 | 0.15 |
|  | (0.12) | (0.12) |  | (0.12) | (0.12) |
| *Education* ^d^ |  |  |  |  |  |
| Medium low | 0.17 | 0.17 |  | -0.04 | -0.04 |
|  | (0.18) | (0.18) |  | (0.22) | (0.22) |
| Medium | 0.24 | 0.24 |  | -0.21 | -0.21 |
|  | (0.19) | (0.19) |  | (0.23) | (0.23) |
| High | 0.25 | 0.25 |  | -0.33 | -0.33 |
|  | (0.18) | (0.18) |  | (0.22) | (0.22) |
| AMR belief: threat (t-1) | 2.44*** | 2.44*** |  |  |  |
|  | (0.18) | (0.18) |  |  |  |
| AMR belief: evidence (t-1) |  |  |  | 1.99*** | 1.99*** |
|  |  |  |  | (0.19) | (0.19) |
|  |  |  |  |  |  |
| Socio-cultural dimension (GAL-TAN) | 0.09 | 0.09 |  | 0.51*** | 0.28 |
|  | (0.16) | (0.42) |  | (0.15) | (0.56) |
| Socio-cultural dimension (GAL-TAN) | -0.41* | -0.41* |  | -0.13 | -0.12 |
|  | (0.19) | (0.19) |  | (0.18) | (0.18) |
|  |  |  |  |  |  |
| Left-media use | 0.11 | 0.13 |  | 0.03 | -0.55 |
|  | (0.43) | (1.09) |  | (0.55) | (1.34) |
| GAL-TAN x media use |  | -0.02 |  |  | 0.79 |
|  |  | (1.39) |  |  | (1.84) |
|  |  |  |  |  |  |
| *N* | 1,249 | 1,249 |  | 1,240 | 1,240 |
| *R^2^* | 0.19 | 0.19 |  | 0.17 | 0.17 |

*Note*. Unstandardized regression coefficients displayed. Robust standard errors in parentheses. All independent and control variables were rescaled to rage from 0 to 1. GAL-TAN = socio-cultural dimension of ideology (“Green-Alternative-Liberal” (GAL) to “Traditional-Authoritarian-Nationalist” (TAN)). AMR = antimicrobial resistance.

a Dependent variable: Agreement to the statement: AMR is one of the greatest threats to public health.

b Dependent variable: Agreement to the statement: Scientific evidence for AMR is weak.

c Reference group: female.

d Reference group: low education.

*** p<0.001, ** p<0.01, * p<0.05
